# Effects of a 12-Week Moderate-to-High Intensity Strength Training Program on the Gait Parameters and Their Variability of Stroke Survivors

**DOI:** 10.3390/brainsci15040354

**Published:** 2025-03-28

**Authors:** Georgios Giarmatzis, Erasmia Giannakou, Ioanna Karagiannakidou, Evangelia Makri, Anna Tsiakiri, Foteini Christidi, Paraskevi Malliou, Konstantinos Vadikolias, Nikolaos Aggelousis

**Affiliations:** 1 Department of Physical Education and Sport Science, Democritus University of Thrace, 69100 Komotini, Thrace, Greece; ggiarmat@phyed.duth.gr (G.G.); egiannak@phyed.duth.gr (E.G.); iokaragi@phyed.duth.gr (I.K.); emakri@phyed.duth.gr (E.M.); pmalliou@phyed.duth.gr (P.M.); 2 Department of Neurology, School of Medicine, Democritus University of Thrace, 68100 Alexandroupolis, Thrace, Greece; atsiakir@med.duth.gr (A.T.); christidi.f.a@gmail.com (F.C.); kvadikol@med.duth.gr (K.V.)

**Keywords:** stroke rehabilitation, gait variability, muscle strengthening, spatiotemporal parameters

## Abstract

Background/Objectives: Chronic stroke survivors often regain walking speed but continue to exhibit heightened gait variability, increasing fall risk. This study investigated the effects of a 12-week moderate-to-high intensity muscle strengthening program on gait parameters and their variability in stroke survivors, without incorporating gait-specific training. Methods: Stroke survivors participated in a twice-weekly, 45–60 min strengthening program using Pilates equipment. Spatiotemporal gait parameters were measured before and after the intervention using 3D motion capture. Walking speed, cadence, step/stride length, step width, and various temporal parameters were analyzed for both paretic and non-paretic limbs, along with their coefficients of variation (CV). Correlation analyses were performed to understand the relationships between parameter changes. Results: Eleven patients (age 61 ± 7.4 years, 9 males) participated in the study. Significant improvements were observed in walking speed for both paretic (0.61 to 0.69 m/s, *p* = 0.032) and non-paretic limbs (0.62 to 0.69 m/s, *p* = 0.024). Step length significantly increased in the paretic limb (0.36 to 0.41 m, *p* = 0.042) with a substantial reduction in variability (CV: 19.91% to 14.99%). Cadence increased significantly in the non-paretic limb (89.24 to 92.01 steps/min, *p* = 0.024). Correlation analysis revealed distinct adaptation patterns between limbs, with speed improvements strongly associated with stride length in both limbs, but with step length only in the non-paretic limb. Conclusions: A moderate-to-high intensity strengthening program, even without direct gait training, can improve walking speed and reduce movement variability in chronic stroke survivors. The intervention predominantly influenced the spatial parameters, with modest changes in the temporal aspects, suggesting that enhanced force production and control primarily affect step execution while preserving temporal gait patterns.

## 1. Introduction

Stroke is a leading cause of disability globally, with approximately 12.2 million new cases each year [1], often resulting in impairments that affect mobility and balance. Therefore, stroke poses a substantial economic burden, with the total cost estimated to be around EUR 45 billion per year in the European Union [2]. Muscle weakness is a common consequence of stroke [3], contributing to altered gait patterns, reduced walking speed [4], and increased energy expenditure [5]. Physical exercise can improve stroke recovery by enhancing walking capabilities, improving muscle strength, cardiovascular fitness, walking speed, endurance, balance, and cognitive function, while also contributing to a better quality of life in stroke patients [6,7]. Even though walking speed is considered the primary biomarker of gait recovery, it has been shown that significant impairments in gait variability remain after exercise [8,9], regardless of the speed increase, predisposing stroke survivors to a greater risk of falling [10]. Therefore, while improving gait speed is a desirable rehabilitation goal, identifying interventions that optimize gait variability is crucial for promoting safe, independent walking post-stroke.

Gait variability refers to the ability to repeat the same gait pattern over multiple walking cycles, indicating better balance control [11] and a more precise regulation of muscle activation [12]. Maintaining the same joint angles across gait strides shows high neurological efficiency, effective proprioceptive feedback, and robust motor planning. Heightened motor variability is a prominent impairment after stroke due to impaired neural pathways [9]. Thus, a highly repeatable walking pattern could mean higher inter-limb coordination [13], physiological cognitive status [14] and less fall risk [15,16] for stroke survivors. As such, one of the primary objectives of stroke rehabilitation should address stroke-related balance deficits [17] by reducing gait variability and improving walking consistency.

The optimal rehabilitation strategies for improving gait variability in stroke patients remain a topic of debate. Both gait retraining exercises and muscle strengthening programs have shown promise in improving overall gait function [18,19]. Gait retraining exercises, such as treadmill training with body weight support or overground walking practice, aim to improve gait quality by reinforcing proper gait patterns and enhancing motor learning [20]. On the other hand, muscle-strengthening programs focus on increasing muscle strength and power, which are essential for generating the forces required for efficient gait [21].

Despite the evidence supporting the use of either gait-retraining or muscle-strengthening exercises in stroke rehabilitation, their relative effectiveness in influencing gait variability remains unclear. Some studies suggest that gait-retraining exercises may be more effective in reducing gait variability, as they directly address its coordination and timing aspects [22,23]. However, other research indicates that increased muscle strength is a significant predictor of functional improvements in daily activities [18,24] and that muscle-strengthening programs can positively affect gait speed and distance [25]. However, much less is known about its effectiveness on gait variability. Villa-Cha et al. showed that strength training enhances the force production steadiness of the knee extensors [26]. However, Patel et al. [27] found that strength training focusing on ankle musculature did not improve gait variability of stroke survivors. Also, a 12-week isolated back muscle strength-training program only slightly managed to improve lumbar sagittal motion steadiness during gait [28]. In the case of the healthy elderly, a 12-week combined exercise program including strength-training exercises managed to decrease gait variability (stride time and length) [29]. Recently, strength-training pilates equipment like the Reformer has been shown to improve trunk control, balance, and gait parameters in patients with chronic stroke, increasing gait speed and step length [30].

Stroke survivors often regain walking speed but continue to exhibit heightened step variability, even when ambulating independently. This persistent gait variability is concerning, as it may increase the risk of falls. The present study aims to evaluate the effects of a high-intensity muscle-strengthening rehabilitation program on gait outcomes and their variability in chronic stroke survivors, without incorporating any functional exercises related to gait. The primary aim was to investigate whether patients attending a supervised program improved in terms of gait parameters and their variability in both paretic and non-paretic sides. A secondary aim was to explore how changes in one parameter related to changes in another, thus identifying how the intervention affected specific gait characteristics for both paretic and non-paretic legs. We hypothesized that a high-intensity muscle-strengthening program, utilizing various Pilates equipment to enhance range of motion and strength, would positively impact gait parameters and variability, with spatial parameters like step and stride length being more affected given their relation to lower-limb muscle force output and the generation of propulsive forces

## 2. Materials and Methods

We conducted a non-randomized trial assessing the effects of a 12-week muscle-strengthening program on a small group of chronic stroke survivors, supplemented with Pilates equipment to promote further strength and flexibility. A wealth of spatiotemporal gait parameters, including speed, step length, step time, and cadence variability, were assessed using advanced biomechanical analysis techniques before and after the intervention.

### 2.1. Participants

The participants were recruited from the outpatient Neurological Rehabilitation Unit at the University Hospital of Alexandroupolis, Greece, between January 2022 and December 2022 through referrals from neurologists and rehabilitation specialists. Participants were included if they met the following criteria: (1) chronic phase of stroke (at least 6 months post-stroke), (2) age above 18 years, (3) walking speed above 0.2 m/s with no upper limit, (4) ability to walk without assistance, and (5) diagnosed hemiparesis with observable motor impairment in the affected limb at the time of enrollment, as confirmed by a licensed healthcare professional. Participants with only non-motor stroke symptoms (such as isolated facial weakness, dysarthria, or sensory deficits) were not eligible for the study. The study sample consisted of eleven participants with a mean age of 61 ± 7.4 years and a BMI of 28 ± 4.24 kg/m^2^. Eight participants presented with left-side paresis, while three had right-side paresis. According to the National Institutes of Health Stroke Scale [31], almost all patients had a score between 1 and 4 (minor stroke), with one patient scoring 7 (moderate stroke).

### 2.2. Intervention

The intervention was conducted twice a week over a 12-week period, with each session lasting 45 to 60 min. Five qualified instructors administered the intervention, with two instructors guiding each stroke survivor through a variety of exercises tailored to individual needs during every session. The intensity of the exercises was monitored using the Borg 1–10 point rating scale, with initial sessions targeting a perceived exertion level of 5–6 (moderate intensity) that progressively increased to 7–8 (high intensity, corresponding to a “really hard” effort) [32] as the participants adapted to the training. The exercises were selected based on the specific needs of each individual. The intensity was progressively increased, taking into account (a) intensity, defined by external load and execution speed, (b) complexity, defined by the number of joints involved and the biomechanical nature of the exercise (closed or open kinetic chain); and (c) functionality of the exercise, defined by position of execution, support surface (stable, unstable), and its resemblance with daily movements. A variety of Pilates equipment was employed, including a reformer tower, wunda chair, armchair, barrel, ring, elastic band, small ball, exercise ball, soft weights, and Bosu.

Each session was structured into three phases:

1. Warm-up phase (5–10 min): involved breathing, posture, and mobility exercises;
2. Main program (35–50 min): comprised of personalized strength exercises targeting the upper and lower body. The duration varied based on individual tolerance and progression;
3. Cool-down (5-min): included breathing exercises and stretching.

### 2.3. Experimental Protocol

Two motion analysis sessions took place, one at the beginning of the training (pre) and one after three months (post). During both sessions, the participants were asked to walk across a 10 m corridor ten times. Markers were placed on specific bony landmarks following the full-body Conventional Gait Model [33], and marker trajectories were recorded with a 10-camera Vicon motion analysis system, with sampling at 100 Hz. Vicon Nexus 2.12.1 ^®^ was used to process all data. Marker trajectories were low-pass filtered at 6 Hz.

### 2.4. Data Analysis

Data analysis was performed by researchers who were blinded to the names of the participants and their functional status, as measured by the NHISS scale, to minimize potential bias in data processing and parameter extraction. The researchers who conducted the intervention were not involved in the data analysis.

Temporal parameters were derived from the identification of key gait events—heel strikes and toe-offs—for both paretic and non-paretic limbs. A gait cycle was defined as the interval between two consecutive ipsilateral heel strikes. Within each gait cycle, stance time was calculated as the duration between heel strike and subsequent toe-off, while swing time represented the period between toe-off and the next heel strike. Step time was measured as the duration between consecutive contralateral heel strikes. Double-support time captured the periods when both feet were in contact with the ground, while single-support time represented phases when only one foot was supporting the body weight.

Spatial parameters were computed using the three-dimensional coordinates of heel markers (LHEE and RHEE). Step length was calculated as the anterior–posterior distance between the heel markers at the moment of heel strike, while stride length represented the anterior–posterior displacement of the same heel marker between consecutive ipsilateral heel strikes. Step width was determined as the mediolateral distance between the heel markers at heel strike events. Walking speed was derived by calculating the anterior–posterior displacement of the RPSI marker (right posterior superior iliac spine) during each gait cycle divided by the cycle duration.

For each participant, data were collected across 10 walking trials. Cadence was computed as the number of steps per minute (120/stride time). The consistency of these parameters was assessed using the coefficient of variation (CV), calculated as the standard deviation divided by the mean and expressed as a percentage. This provided a standardized measure of variability that could be compared across different parameters and between paretic and non-paretic limbs. To estimate the effect of the intervention, the Hedges’ g effect size for paired samples with a small sample size correction was calculated for each variable [34]. Effect sizes were classified as small (d = 0.2), medium (d = 0.5), and large (d = 0.8). To evaluate significant differences between the pre- and post-sessions, a Wilcoxon paired *t*-test was performed for the average and CV values.

Correlation analyses between gait parameters were visualized using heatmaps to examine the relationships between percentage changes in the spatiotemporal parameters following the intervention. For each parameter, percentage changes were calculated as the difference between the post- and pre-intervention values normalized to pre-intervention values. Pearson correlation coefficients were computed for all parameter pairs separately for paretic and non-paretic limbs, resulting in two correlation matrices.

All calculations were performed using custom scripts in Python 3.8, ensuring standardized processing across all participants and trials.

## 3. Results

Average values of all of the spatiotemporal parameters for both paretic and non-paretic sides, across the pre- and post-training sessions, are reported in Table 1, along with the corresponding variability (CV%).

**Figure 1:**
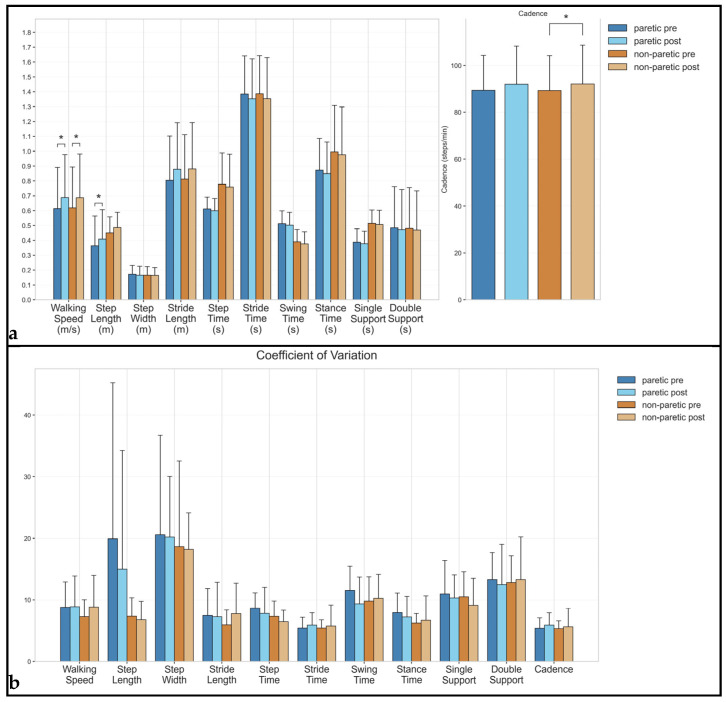
Spatiotemporal gait parameters in stroke survivors before and after rehabilitation. (**a**) Mean values (±standard deviation) are shown for the paretic and non-paretic limbs during pre- and post-intervention assessments. Left panel shows spatial parameters (m) and temporal parameters (s); right panel shows cadence (steps/min). Parameters are grouped by units with appropriate scales. Asterisks indicate statistically significant differences between pre- and post-intervention values (*p* < 0.05). (**b**) CV values (±standard error) are shown as percentages for both paretic and non-paretic limbs during pre- and post-intervention assessments. Lower CV values indicate reduced variability and improved consistency in gait parameters. Asterisks indicate statistically significant differences between pre- and post-intervention values (*p* < 0.05).

An analysis of spatiotemporal gait parameters revealed varying degrees of change following the intervention. The results are presented by parameter category, with the values expressed as the mean (range), and the statistical significance determined at *p* < 0.05 (see Figure 1).

### 3.1. Spatiotemporal Parameters

Walking speed showed significant improvements bilaterally, increasing from 0.61 (0.20–1.13) m/s to 0.69 (0.20–1.20) m/s in the paretic limb (*p* = 0.032, Hedges’ g = 0.25) and from 0.62 (0.20–1.14) m/s to 0.69 (0.19–1.21) m/s in the non-paretic limb (*p* = 0.024, Hedges’ g = 0.23). Movement variability remained relatively stable in the paretic limb (CV: 8.75% to 8.84%) but showed a small increase in the non-paretic limb (CV: 7.26% to 8.78%).

Cadence increased significantly in the non-paretic limb, from 89.24 (60.79–112.62) steps/min to 92.01 (59.24–118.93) steps/min (*p* = 0.024, Hedges’ g = 0.17) with a similar, though non-significant, trend in the paretic limb (89.35 to 91.92 steps/min). Cadence variability did not change in both limbs (paretic: CV: 5.37% to 5.91%; non-paretic: CV: 5.31% to 5.61%).

### 3.2. Spatial Parameters

Step length showed significant improvement in the paretic limb from 0.36 (0.04–0.71) m to 0.41 (0.07–0.74) m (*p* = 0.042, Hedges’ g = 0.21), while the non-paretic limb increased from 0.45 (0.33–0.71) m to 0.49 (0.35–0.72) m. The corresponding variability showed a notable reduction in the paretic limb (CV: 19.91% to 14.99%, Hedges’ g = −0.21) and non-paretic limb (CV: 7.33% to 6.79%, Hedges’ g = −0.18). The step width remained relatively stable bilaterally (paretic: 0.17 to 0.17 m; non-paretic: 0.17 to 0.16 m), with minimal changes in variability (paretic: CV: 20.55% to 20.18%; non-paretic: CV: 18.64% to 18.19%). Stride length showed increases in both limbs (paretic: 0.80 to 0.88 m; non-paretic: 0.81 to 0.88 m). Its variability remained stable in the paretic limb (CV: 7.47% to 7.25%) but increased in the non-paretic limb (CV: 5.94% to 7.77%).

### 3.3. Temporal Parameters

Step time showed small reductions bilaterally (paretic: 0.61 to 0.60 s; non-paretic: 0.78 to 0.76 s), with decreased variability in both limbs (paretic: CV: 8.63% to 7.80%; non-paretic: CV: 7.32% to 6.46%). Stride time decreased slightly and almost significantly in the non-paretic limb (1.39 to 1.35 s, *p* = 0.054, Hedges’ g = −0.12), with a similar trend in the paretic limb (1.38 to 1.35 s). Variability showed small increases in both limbs (paretic: CV: 5.41% to 5.91%; non-paretic: CV: 5.41% to 5.76). Stance time demonstrated modest reductions in both limbs (paretic: 0.87 to 0.85 s; non-paretic: 1.00 to 0.98 s). Variability decreased in the paretic limb (CV: 7.94% to 7.21%) but increased slightly in the non-paretic limb (CV: 6.24% to 6.72%).

Swing time did not change bilaterally (paretic: 0.51 to 0.50 s, Hedges’ g = −0.11; non-paretic: 0.39 to 0.38 s, Hedges’ g = −0.16). Variability decreased in the paretic limb (CV: 11.53% to 9.34%) but increased slightly in the non-paretic limb (CV: 9.80% to 10.23%). Single-support time remained relatively stable (paretic: 0.39 to 0.38 s; non-paretic: 0.51 to 0.51 s), with decreased variability in both limbs (paretic: CV: 10.94% to 10.28%; non-paretic: CV: 10.47% to 9.10%). Double-support time showed minimal changes (paretic: 0.48 to 0.47 s, Hedges’ g = −0.04; non-paretic: 0.48 to 0.47 s), with slightly decreased variability in the paretic limb (CV: 13.31% to 12.47%) and a small increase in the non-paretic limb (CV: 12.82% to 13.30%).

#### Correlation Analysis

**Figure 2:**
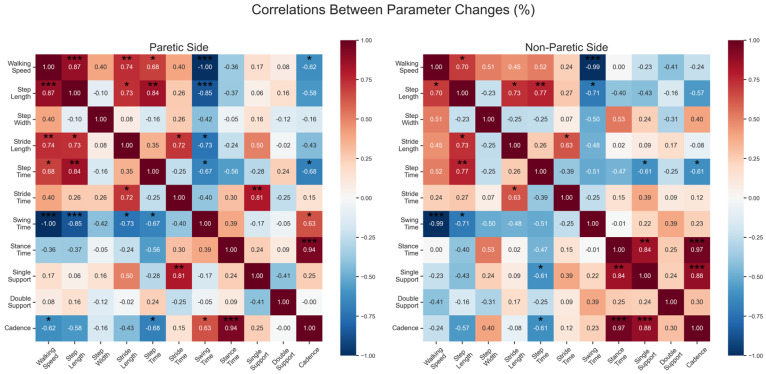
Correlation heatmaps showing relationships between percentage changes in spatiotemporal parameters for paretic (**left**) and non-paretic (**right**) sides following intervention. Correlation coefficients range from −1.00 (dark blue) to 1.00 (dark red). Positive correlations indicate parameters changing in the same direction (both increasing or both decreasing), while negative correlations indicate parameters changing in opposite directions. The correlation matrix is symmetric around the diagonal. The size and color intensity of each cell represent the strength of the correlation, with darker colors indicating stronger relationships. * *p* < 0.05, ** *p* < 0.01, *** *p* < 0.001.

Changes in the spatiotemporal parameters demonstrated distinct correlation patterns between the paretic and non-paretic sides following intervention (Figure 2). Detailed presentation of the stronger relations (r > 0.5) are shown in Appendix A—Figure A1. Almost absolute correlation between stride time and cadence was expected due to their inverse relationship [35]. On the paretic side, improvements in walking speed were strongly associated with increases in stride length (r = 0.95, *p* < 0.001) and moderately with cadence increases (r = 0.63, *p* < 0.05) but showed no significant correlation with step length (r = 0.17, *p* > 0.05). Strong correlations emerged among the temporal parameters, with stride time changes strongly correlating with both stance time (r = 0.84, *p* < 0.01) and double-support (r = 0.74, *p* < 0.01) changes, as expected. As was also anticipated, cadence changes showed strong negative correlations with stance time (r = −0.85, *p* < 0.001) and double-support (r = −0.74, *p* < 0.01) changes. Single-support time changes demonstrated strong positive correlations with step length changes (r = 0.80, *p* < 0.01), suggesting coordinated improvements in weight bearing and step execution on the paretic side.

The non-paretic side exhibited a different correlation pattern. Walking speed improvements maintained strong positive correlations with both stride length (r = 0.96, *p* < 0.001) and step length (r = 0.87, *p* < 0.001) changes, indicating a more integrated spatial adaptation compared to the paretic side. While the relationship between stride time and stance time changes remained strong (r = 0.76, *p* < 0.01), the correlations among the other temporal parameters were generally weaker compared to the paretic side. Double-support time changes demonstrated moderate-to-strong negative correlations with the spatial parameters, particularly with step length changes (r = −0.74, *p* < 0.01).

Step width changes showed relatively weak correlations with most parameters on both sides (r ranging from −0.42 to 0.40), suggesting that mediolateral step adjustments were relatively independent of other gait parameter changes.

## 4. Discussion

This study investigated the effects of a 12-week moderate-to-high intensity strengthening program on gait parameters and their variability in chronic stroke survivors. To our knowledge, our study is the first to report between-leg differences in gait variability influenced by a training program. Our findings revealed three key patterns of adaptation. First, the intervention predominantly influenced the spatial parameters of gait, with notable improvements in stride length driving increases in walking speed, while the temporal parameters showed more modest changes. Second, movement variability demonstrated selective improvements, particularly in the step length consistency of the paretic limb, suggesting enhanced motor control in specific aspects of the gait cycle. Third, a correlation analysis revealed distinct relationships between the gait parameters in the paretic and non-paretic limbs, indicating different mechanisms of adaptation between sides. These findings suggest that strength training, even without direct gait-specific training, can positively influence walking patterns in chronic stroke survivors through multiple mechanisms.

The most prominent finding was the differential effect of the intervention on the spatial versus temporal parameters of gait. Walking speed showed significant improvements bilaterally (0.61 to 0.69 m/s in the paretic limb, *p* = 0.032; 0.62 to 0.69 m/s in the non-paretic limb, *p* = 0.024), driven by increases in both step/stride length and cadence. Notably, the paretic limb demonstrated significant improvements in step length (0.36 to 0.41 m, *p* = 0.042) accompanied by a substantial reduction in variability (CV: 19.91% to 14.99%), suggesting enhanced motor control in spatial positioning. Step width remained consistent (approximately 0.17 m bilaterally) with unchanging variability patterns (CV ~20%), indicating preserved lateral stability control. These findings suggest that enhanced force production capacity, particularly in the lower-limb musculature, enabled participants to take longer steps while maintaining stable mediolateral control. The improvement in spatial parameters aligns with previous research showing that muscle-strengthening interventions primarily enhance force production capabilities [36,37,38,39], which directly influence the ability to generate longer steps. The relative stability of the temporal parameters, including stance time, swing time, and double-support time, suggests that the underlying timing of the gait cycles remained largely unchanged, while the spatial components adapted to increased force production capacity. The non-paretic limb also showed significant improvement in cadence (*p* = 0.024), albeit with a small effect size (g = 0.17). The similarity in pre- and post-intervention cadence (see Table 1) between limbs suggests that our intervention had relatively balanced effects on temporal parameters bilaterally.

A particularly noteworthy finding was the selective improvement in movement variability following the intervention. The most substantial reduction in variability was observed in the step length of the paretic limb, where CV decreased markedly from 19.91% to 14.99%, whereas the non-paretic limb maintained relatively low variability throughout (CV: 7.33% to 6.79%). This asymmetric improvement in spatial consistency suggests that strengthening exercises enhanced not only force production capacity but also the precision of force production and movement control in the paretic limb, as seen in previous research [40,41]. In contrast to the improvements in spatial variability, the temporal parameters showed mixed responses in terms of movement consistency. A notable finding was the persistently high step width variability across both limbs (~20%), which remained unchanged despite improvements in other parameters. These values, substantially higher than typical healthy adult values (~7–10%) [42], likely reflect underlying central nervous system deficits in lateral stability control that may be more resistant to strength-focused interventions. The maintenance of high step width variability suggests that, while our intervention successfully improved anteroposterior force production and control (as evidenced by improved step length parameters), it had a limited impact on the neural control mechanisms responsible for lateral stability during gait. This finding aligns with previous research suggesting that lateral stability control may be mediated by separate neural pathways than those in the fore–aft direction during walking [43]. Mediolateral stability during gait relies on complex neurophysiological systems beyond mere muscle strength, including proprioceptive awareness, visual feedback integration, vestibular function, and coordinated motor control networks. These systems often sustain significant damage following stroke, creating deficits that resistance training alone cannot fully address. While our strength-training intervention improved force generation capacity, it did not specifically target hip abductor and adductor musculature, which play a crucial role in mediolateral stability. Last, according to the literature [44,45,46], the intricate sensorimotor integration required for improved dynamic balance control during walking seems to require more targeted interventions than resistance training to specifically challenge these neurological systems.

The correlation analysis revealed distinct patterns in the parameter relationships between the paretic and non-paretic limbs. On the **paretic side**, improvements in walking speed were strongly associated with increases in stride length (r = 0.95, *p* < 0.001) and moderately with cadence increases (r = 0.63, *p* < 0.05). Strong correlations emerged among the temporal parameters, with stride time changes strongly correlating with both stance time (r = 0.84, *p* < 0.01) and double-support (r = 0.74, *p* < 0.01) changes. Notably, single-support time changes demonstrated strong positive correlations with step length changes (r = 0.80, *p* < 0.01). These results suggest that alterations in overall gait timing are closely linked to changes in the weight-bearing phases, indicating a coordinated adjustment in both temporal gait dynamics and step execution on the paretic side. The **non-paretic side** exhibited a different correlation pattern, suggesting more flexible control strategies. Walking speed improvements maintained strong positive correlations with both stride length (r = 0.96, *p* < 0.001) and step length (r = 0.87, *p* < 0.001) changes, indicating a more integrated spatial adaptation compared to the paretic side. While the relationship between stride time and stance time changes remained strong (r = 0.76, *p* < 0.01), the correlations among the other temporal parameters were generally weaker compared to the paretic side. These correlation patterns indicate that both limbs achieved similar functional outcomes (improved walking speed), however, through different control strategies.

Several limitations of this study warrant consideration when interpreting our findings. First, the small sample size (*n* = 11) and lack of a control group limit the generalizability of our results and preclude definitive causal relationships between the intervention and the observed changes. Second, while our intervention focused on strengthening exercises, we did not directly measure muscle strength or force production capacity, making it difficult to establish direct links between strength gains and gait improvements. The heterogeneity in participants’ initial impairment levels and time since stroke may have influenced individual responses to the intervention, though this variation reflects the clinical reality of stroke rehabilitation. Third, despite the well-established relationship between gait variability and fall risk, we did not directly assess fall occurrence or fall-risk metrics during or after the intervention period. Future studies should incorporate prospective fall monitoring and standardized fall risk assessments to determine whether improvements in gait parameters and reduced variability translate to clinically meaningful reductions in fall events. Such data would strengthen the clinical relevance of our findings and provide more direct evidence for rehabilitation practices aimed at fall prevention. Finally, while our analysis focused on spatiotemporal parameters, we did not include kinematic or kinetic measurements that could have provided deeper insights into the biomechanical mechanisms underlying the observed changes.

While the Hedges’ g effect sizes observed in our study were relatively small (ranging from 0.21 to 0.25 for the key parameters), it is important to consider these values in the context of chronic stroke rehabilitation. The participants in our study were in the chronic phase of stroke recovery (at least 6 months post-stroke), when spontaneous neurological recovery has typically plateaued and interventional gains tend to be more modest. Even small improvements in gait parameters at this stage may translate to meaningful functional benefits for patients. For example, the average improvement in walking speed of 0.08 m/s approaches the minimal clinically important difference (MCID) of 0.10 m/s established for pathological populations [47]. Similarly, the 25% reduction in step length variability in the paretic limb could potentially contribute to improved stability and reduced fall risk.

## 5. Conclusions

Our findings demonstrate that a strengthening program using Pilates equipment can positively influence gait parameters in chronic stroke survivors through multiple mechanisms, primarily by enhancing the spatial aspects of gait while preserving temporal control strategies. The intervention’s differential effects on paretic and non-paretic limbs, coupled with distinct patterns of movement variability adaptation, suggest that force production capabilities may be a crucial foundation for gait recovery post-stroke. These results highlight the potential value of incorporating intensive strength training in stroke rehabilitation, even in the chronic phase, and suggest that improvements in walking function can be achieved without direct gait-specific training. However, little is known about the individual muscle recruitment strategy to accomplish the outcomes reported in this study. It has been known that stroke patients respond differently to the same training protocol, given their unique characteristics of neuromuscular deficiencies and the respective potential for change [48]. Future work from our lab will investigate the changes in muscle force output as a result of the training protocol and elucidate the underlying reasons for this study’s conclusions. Understanding these relationships through a comprehensive biomechanical analysis would provide valuable insights into developing more effective, personalized rehabilitation approaches for stroke survivors. Finally, future studies should incorporate patient-reported outcome measures to better understand the subjective impact of interventions on daily functioning and quality of life. Measures such as the Stroke Impact Scale, the Activity-specific Balance Confidence Scale, or PROMIS Physical Function could provide valuable insights into how improvements in gait parameters translate to meaningful changes in patients’ daily activities and perceived capabilities.

## Figures and Tables

**Table 1:** Mean (range) and CV (range) for all spatiotemporal variables during gait for both paretic and non-paretic sides, pre- and post-intervention. Hedges’ g values show clinically important differences, and *p* values denote statistically significant differences.

Parameter	Leg	Mean Pre (Range)	Mean Post (Range)	Hedges’ g	Mean *p*-Value	CV Pre (Range)	CV Post (Range)	CV Hedges’ g	CV *p*-Value
Spatiotemporal Parameters									
Walking Speed	paretic	0.61 (0.20–1.13)	0.69 (0.20–1.20)	0.25	0.032	8.75 (4.09–18.47)	8.84 (3.05–17.63)	0.02	0.765
	non-paretic	0.62 (0.20–1.14)	0.69 (0.19–1.21)	0.23	0.024	7.26 (3.12–12.15)	8.78 (3.83–20.59)	0.35	0.465
Cadence	paretic	89.35 (60.65–112.91)	91.92 (60.27–119.23)	0.16	0.147	5.37 (3.59–8.40)	5.91 (3.60–9.98)	0.28	0.365
	non-paretic	89.24 (60.79–112.62)	92.01 (59.24–118.93)	0.17	0.024	5.31 (3.33–7.51)	5.61 (2.92–13.22)	0.12	1.000
Spatial Parameters									
Step Length	paretic	0.36 (0.04–0.71)	0.41 (0.07–0.74)	0.21	0.042	19.91 (3.53–90.83)	14.99 (3.11–71.11)	−0.21	0.102
	non-paretic	0.45 (0.33–0.71)	0.49 (0.35–0.72)	0.33	0.083	7.33 (3.16–11.64)	6.79 (3.73–13.10)	−0.18	0.898
Step Width	paretic	0.17 (0.07–0.24)	0.17 (0.07–0.25)	−0.10	0.765	20.55 (5.95–52.27)	20.18 (6.57–32.90)	−0.03	0.700
	non-paretic	0.17 (0.07–0.24)	0.16 (0.07–0.25)	−0.02	0.966	18.64 (7.58–53.76)	18.19 (6.67–27.16)	−0.04	0.898
Stride Length	paretic	0.80 (0.32–1.40)	0.88 (0.28–1.46)	0.23	0.102	7.47 (2.45–18.94)	7.25 (2.15–22.71)	−0.04	0.966
	non-paretic	0.81 (0.32–1.42)	0.88 (0.28–1.47)	0.21	0.175	5.94 (2.16–9.11)	7.77 (3.39–20.33)	0.45	0.206
Temporal Parameters									
Step Time	paretic	0.61 (0.49–0.72)	0.60 (0.42–0.70)	−0.15	0.413	8.63 (5.19–11.71)	7.80 (2.92–16.77)	−0.23	0.320
	non-paretic	0.78 (0.55–1.30)	0.76 (0.51–1.35)	−0.08	0.365	7.32 (4.13–13.65)	6.46 (3.21–9.26)	−0.37	0.175
Stride Time	paretic	1.38 (1.06–1.98)	1.35 (1.01–2.00)	−0.12	0.206	5.41 (3.54–8.75)	5.91 (3.62–9.53)	0.25	0.365
	non-paretic	1.39 (1.07–1.98)	1.35 (1.01–2.03)	−0.12	0.054	5.41 (3.31–7.97)	5.76 (2.89–14.66)	0.13	0.898
Stance Time	paretic	0.87 (0.66–1.30)	0.85 (0.61–1.31)	−0.10	0.175	7.94 (4.23–14.58)	7.21 (4.09–14.72)	−0.21	0.278
	non-paretic	1.00 (0.68–1.73)	0.98 (0.61–1.76)	−0.06	0.240	6.24 (3.95–9.21)	6.72 (3.14–17.23)	0.15	0.700
Swing Time	paretic	0.51 (0.40–0.68)	0.50 (0.38–0.69)	−0.11	0.320	11.53 (4.84–17.03)	9.34 (4.42–18.30)	−0.51	0.365
	non-paretic	0.39 (0.24–0.49)	0.38 (0.23–0.48)	−0.16	0.175	9.80 (5.03–16.76)	10.23 (4.97–18.08)	0.11	0.577
Single Support	paretic	0.39 (0.22–0.50)	0.38 (0.24–0.48)	−0.11	0.365	10.94 (4.77–22.40)	10.28 (5.01–16.46)	−0.14	0.966
	non-paretic	0.51 (0.41–0.69)	0.51 (0.36–0.73)	−0.08	0.638	10.47 (5.64–15.52)	9.10 (3.56–18.30)	−0.31	0.365
Double Support	paretic	0.48 (0.22–1.06)	0.47 (0.14–1.04)	−0.04	0.240	13.31 (7.85–23.29)	12.47 (3.55–25.41)	−0.15	0.520
	non-paretic	0.48 (0.21–1.04)	0.47 (0.14–1.03)	−0.04	0.147	12.82 (9.28–24.06)	13.30 (3.31–25.93)	0.08	0.898

**Figure A1:**
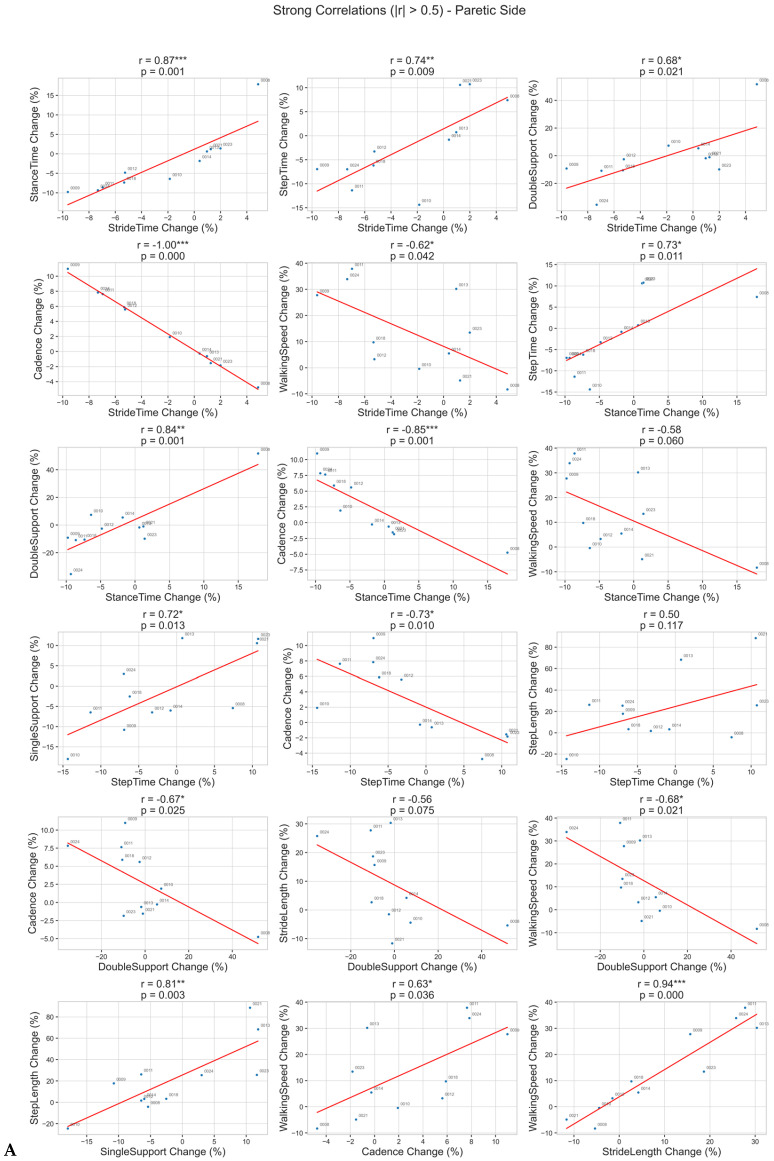

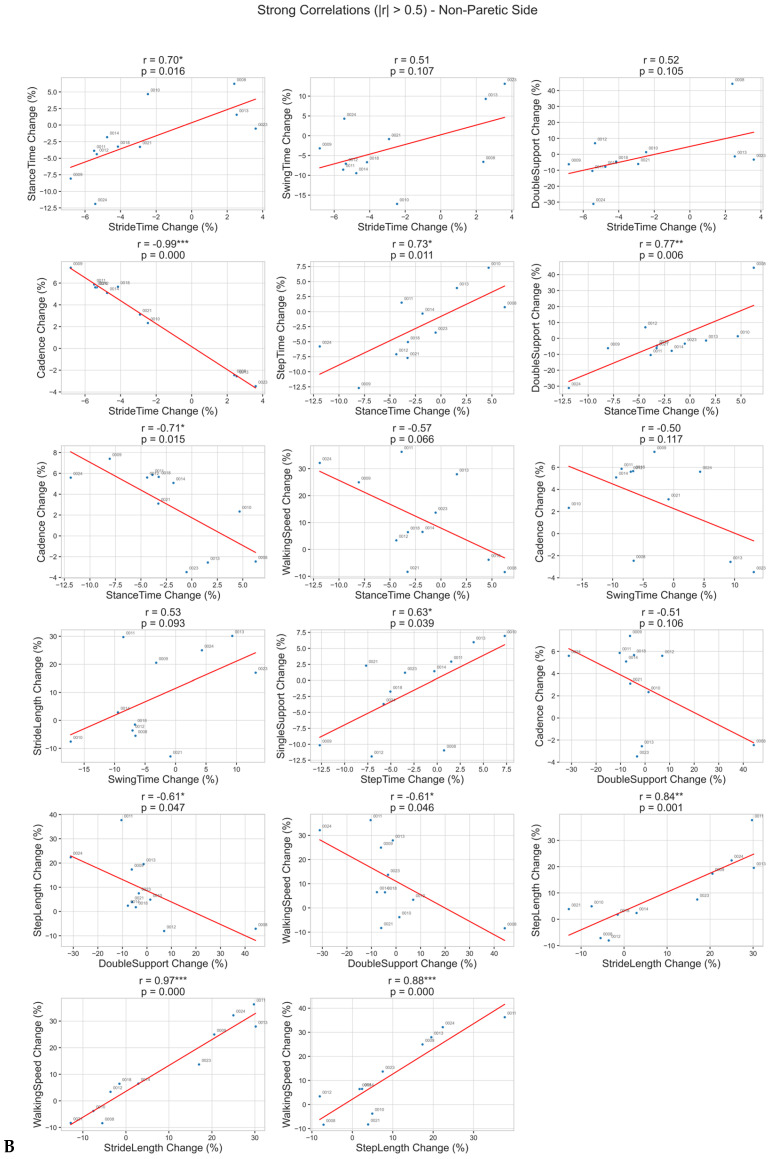
Strong correlations (|r| > 0.5) between percentage changes in spatiotemporal parameters following a 12-week intervention, shown separately for paretic (**A**) and non-paretic (**B**) sides. Each scatter plot represents a parameter pair with correlation coefficient |r| > 0.5, with individual subjects marked by their ID numbers. Red lines indicate linear regression fits. Statistical significance is denoted by asterisks (* *p* < 0.05, ** *p* < 0.01, *** *p* < 0.001).

## Data Availability

The data presented in this study are available upon request from the corresponding author. The data are not publicly available due to privacy reasons.
